# A Precise Oxide Film Thickness Measurement Method Based on Swept Frequency and Transmission Cable Impedance Correction

**DOI:** 10.3390/s25020579

**Published:** 2025-01-20

**Authors:** Yifan Li, Qi Xiao, Lisha Peng, Songling Huang, Chaofeng Ye

**Affiliations:** 1 School of Information Science and Technology, ShanghaiTech University, Shanghai 201210, China; liyf12022@shanghaitech.edu.cn (Y.L.); xiaoqi1@shanghaitech.edu.cn (Q.X.); 2 Department of Electrical, Tsinghua University, Beijing 100084, China; penglisha@mail.tsinghua.edu.cn (L.P.); huangsling@tsinghua.edu.cn (S.H.)

**Keywords:** eddy current testing (ECT), transmission line model, impedance correction, oxide film thickness measurement, swept frequency

## Abstract

Accurately measuring the thickness of the oxide film that accumulates on nuclear fuel assemblies is critical for maintaining nuclear power plant safety. Oxide film thickness typically ranges from a few micrometers to several tens of micrometers, necessitating a high-precision measurement system. Eddy current testing (ECT) is commonly employed during poolside inspections due to its simplicity and ease of on-site implementation. The use of swept frequency technology can mitigate the impact of interference parameters and improve the measurement accuracy of ECT. However, as the nuclear assembly is placed in a pool for inspection, a cable several dozen meters in length is used to connect the ECT probe to the instrument. The measurement is affected by the transmission line and its effect is a function of the operating frequencies, resulting in errors for swept frequency measurements. This paper proposes a method for precisely measuring oxide film thickness based on the swept frequency technique and long transmission line impedance correction. The signals are calibrated based on a transmission line model of the cable, effectively eliminating the influence of the transmission cable. A swept frequency signal-processing algorithm is developed to separate the parameters and calculate oxide film thickness. To verify the feasibility of the method, measurements are conducted on fuel cladding samples with varying conductivities. It is found that the method can accurately assess oxide film thickness with varying conductivity. The maximum error is 3.42 μm, while the average error is 1.82 μm. The impedance correction reduces the error by 66%. The experimental results indicate that this method can eliminate the impact of long transmission cables, and the algorithm can mitigate the influence of material conductivity. This method can be utilized to measure oxide film thickness in nuclear power maintenance inspections following extensive testing and engineering optimization.

## 1. Introduction

A nuclear fuel assembly typically consists of hundreds of fuel rods with zirconium alloy cladding. These fuel rods serve as the first barrier to radioactive fission products. In the operating environment, fuel rods are subjected to prolonged neutron irradiation, high pressure (≥15.5 MPa), high temperatures (300–500 °C), and erosion and corrosion caused by high-flow-rate circulating water. A slow oxidation reaction occurs on the cladding surface, gradually forming an oxide layer. This oxide film not only increases the thermal resistance between the cladding surface and the coolant but also significantly compromises the heat transfer capacity of the fuel assembly. The increased thermal resistance leads to a rise in the temperature of the fuel rods, accelerating the corrosion rate and posing substantial risks to the reactor’s safe operation [1,2]. To mitigate these risks, once the allowable oxide film thickness on the cladding is reached, the fuel assembly must be decommissioned and replaced. Therefore, accurately measuring the oxide film thickness on fuel rods is of critical importance for ensuring the safety, efficiency, and economic performance of nuclear reactors [3,4].

Currently, various methods are employed to measure oxide film thickness, including eddy current testing (ECT) [5,6,7,8], optical testing [9], high-frequency ultrasonic testing [10], and terahertz testing [11]. Among these, ECT stands out due to its low cost, high measurement accuracy, and rapid detection speed, making it a preferred choice for micron-scale oxide film thickness measurement in poolside inspections [12,13]. The core principle of ECT lies in the interaction between an induction coil and the conductive sample, with the impedance of the coil being a function of the distance between the two. However, the output signal of ECT is influenced not only by oxide film thickness but also by material properties such as permeability and conductivity [14,15]. In practical applications, these material properties are often unknown or may change over time, complicating the measurement process. Therefore, to ensure accurate measurements, it is critical to decouple the effects of material conductivity from thickness measurements [16].

The impedance of an induction coil is a function of the distance between the coil and the conductive sample, which is the fundamental principle behind measuring oxide film thickness using ECT [17]. In recent years, researchers have explored various strategies to enhance the accuracy of ECT-based thickness measurements. For instance, A. Sethuraman et al. proposed a table lookup method based on impedance curves to determine the thickness and conductivity of metal coatings [18]. V.A. Syas’ko et al. introduced a calibration method that accounted for both geometric and electromagnetic parameters to improve measurement precision [19]. Shoya Adachi et al. proposed a sensor configuration comprising anisotropic magnetoresistance (AMR) sensors and induction coils, which effectively quantified the thickness of steel sheet piles at the millimeter scale [20]. Grzegorz Tytko et al. suggested a pot core sensor configuration for quantifying the thickness of thermal barrier coatings [21]. X. Meng et al. introduced a dual-coil sensor architecture with an iterative algorithm to enhance ECT efficacy, achieving a 3% error margin in millimeter-level measurements [22]. W. Cheng et al. proposed a swept frequency ECT method that utilized standardized impedance and the maximum phase angle to measure oxide film thicknesses ranging from several hundred microns to a few millimeters [23,24]. Y. Li et al. employed a coil sensor to assess the thickness of thermal barrier coatings, achieving a maximum inaccuracy of approximately 20 μm [25]. W. Guo et al. developed a high-frequency ECT sensor specifically designed for evaluating TBC thickness, providing further improvements in measurement precision [26]. J. Xu et al. proposed a correction method for the coupling between coils and conductors, and used frequencies as high as tens of MHz for micrometer-level thickness measurements [27]. C. Wang et al. proposed a dual-coil eddy current sensor for measuring the thickness of metal thin films, further broadening the scope of ECT applications [28]. These advancements have significantly improved ECT performance for measuring oxide and thermal barrier coatings.

ECT sensors typically operate in the frequency range of up to several MHz, and the influence of transmission cables on the sensor output becomes a critical issue. However, a thorough review of the literature reveals that the impact of transmission cables on ECT measurements has not been systematically investigated. In this work, we address this issue by analyzing the effect of transmission cables on high-frequency ECT sensor output. This paper proposes a novel correction method to minimize signal distortion caused by transmission cables, thereby improving the accuracy of oxide film thickness measurements. This investigation aims to fill a critical gap in the literature and enhance the practical applicability of ECT for non-destructive testing in nuclear engineering.

## 2. Principle

**Figure 1:**
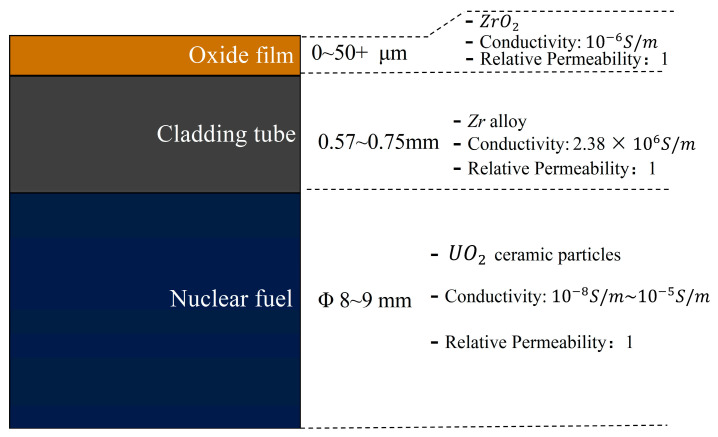
Typical structure of a fuel rod with an oxide film.

The typical structure of a fuel rod is illustrated in Figure 1. The nuclear fuel is non-conductive, rendering its effect in ECT negligible. The material of the cladding is Zr alloy, with a conductivity and relative permeability approximately 1.6 × 10^6^ S/m and 1, respectively. The thickness of the tube ranges from 0.57 to 0.75 mm. The thickness of the oxide film typically ranges from a few micrometers to several tens of micrometers. The oxide film on the fuel rods is primarily composed of ZrO_2_, which is known to be non-conductive in the low-temperature range (room temperature to 600 °C) [29]. Both the laboratory experiments conducted at room temperature and the poolside inspections, typically carried out at temperatures ranging from approximately 25 °C to 60 °C, occur in a temperature range far below the threshold where ZrO_2_ exhibits a significant increase in electrical conductivity. Therefore, the oxide film can be considered electrically insulating under these conditions.

The principle of ECT is based on the mutual inductance effect between a coil and a conductive material. By applying alternating current (AC) to the excitation coil, an alternating magnetic field is generated around the coil. When a fuel rod is placed within this alternating magnetic field, eddy currents are induced in the fuel rod. The distance between the coil and the conductive material affects the intensity and distribution of the induced currents, leading to variations in the impedance of the coil. Therefore, the oxide film thickness can be measured by monitoring the impedance of the coil.

### 2.1. Equivalent Circuit Model

**Figure 2:**
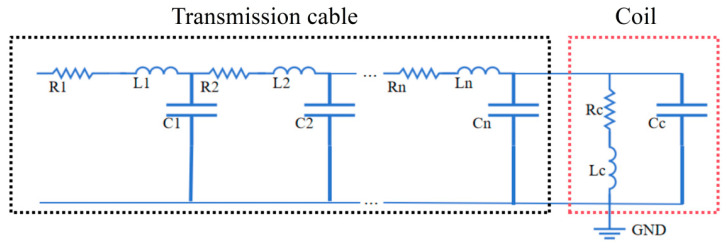
Equivalent circuit model of a coil and transmission cable.

In a typical ECT setting, the coil is connected to the instrument via a coaxial cable. The equivalent circuit of an ECT coil and the cable linking the coil to the instrument is shown in Figure 2. Here Lc denotes the coil inductance, Cc represents the coil capacitance, and Rc indicates the resistance of the coil. The impact of the cable is analyzed using a distributed circuit model, where the cable is treated as a finite periodic structure composed of a series and parallel combination of resistors, inductors, and capacitors. It is assumed that the cable consists of n units. Ri, Li, and Ci (where i = 1, 2, 3… n) in the figure represent the resistance, inductance, and capacitance of the ith unit, respectively.

The impedance of the coil (*Z_c_*) is written as (1), where *ω* is the angular frequency of the alternating current:

(1) $$Z_{C}=(R_{C}+j\omega L_{C})∥(\frac{1}{j\omega Cc})=\frac{\frac{1}{j\omega Cc}\times (Rc+j\omega Lc)}{\frac{1}{j\omega Cc}+(Rc+j\omega Lc)}$$

The impedances $Z_{1^{\prime }}$ and $Z_{2^{\prime }}$ are defined as (2) and (3), respectively [30]:

(2) $$Z_{1^{\prime }}=R_{i}+jwL_{i}$$

(3) $$Z_{2^{\prime }}=\frac{1}{jwC_{i}}$$

The impedance seen at the left-hand side of the nth unit ($Z_{n}$) is written as (4). Then, the impedance seem at the left-hand side of the (*n* − 1)th unit ($Z_{n-1}$) is written as (5). The calculation is repeated n times to obtain the impedance of the coil and the cable ($Z_{1}$) seen by the instrument, as written in (6).

(4) $$Z_{n}=Z_{c}∥Z_{2^{\prime }}+Z_{1^{\prime }}=\frac{Z_{c}\cdot Z_{2^{\prime }}}{Z_{c}+Z_{2^{\prime }}}+Z_{1^{\prime }}$$

(5) $$Z_{n-1}=Z_{n}∥Z_{2^{\prime }}+Z_{1^{\prime }}=\frac{Z_{n}\cdot Z_{2^{\prime }}}{Z_{n}+Z_{2^{\prime }}}+Z_{1^{\prime }}$$

(6) $$Z_{1}=Z_{2}∥Z_{2^{\prime }}+Z_{1^{\prime }}=\frac{Z_{2}\cdot Z_{2^{\prime }}}{Z_{2}+Z_{2^{\prime }}}+Z_{1^{\prime }}$$

### 2.2. Reversely Calculation

**Figure 3:**
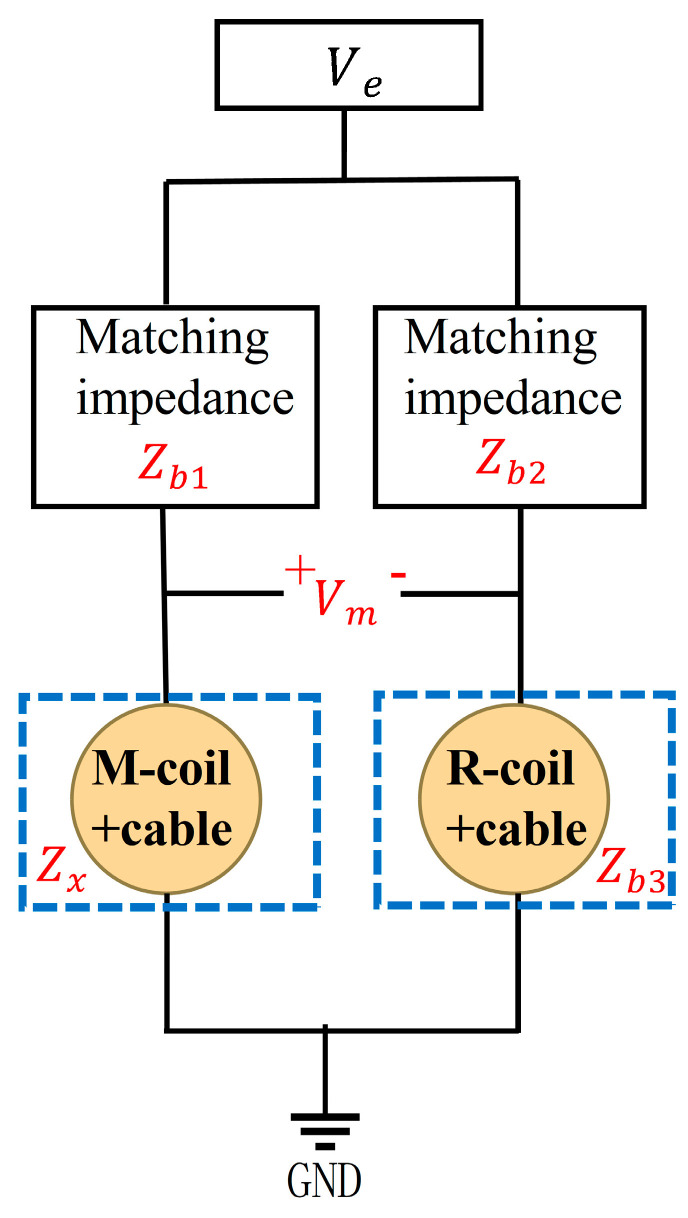
A schematic diagram of the measurement circuit.

The schematic diagram of the measuring circuit is shown in Figure 3. The measurement circuit comprises a bridge circuit, which should be balanced to enhance its sensitivity. Ideally, the impedances in the bridge circuit are equal after balancing [31], denoted as $Z_{0}$, as shown in (7):

(7) $${Z_{0}=Z}_{b1}=Z_{b2}=Z_{b3}=Z_{x}$$

The output signal of the bridge circuit is denoted as $V_{m}$. To eliminate the influence of the transition cable, it is required to infer the impedance of the coil from the measured $V_{m}$. $V_{m\;}$ is written as (8), where $V_{e}$ is the excitation voltage applied to the bridge. ${\;Z}_{x\;}$ is written as (9).

(8) $$V_{m}=(\frac{Z_{x}}{Z_{x}+Z_{0}}-0.5)\cdot V_{e}$$

(9) $$Z_{x}=Z_{0}\cdot \frac{V_{m}+0.5V_{e}}{0.5V_{e}-V_{m}}$$

**Figure 4:**
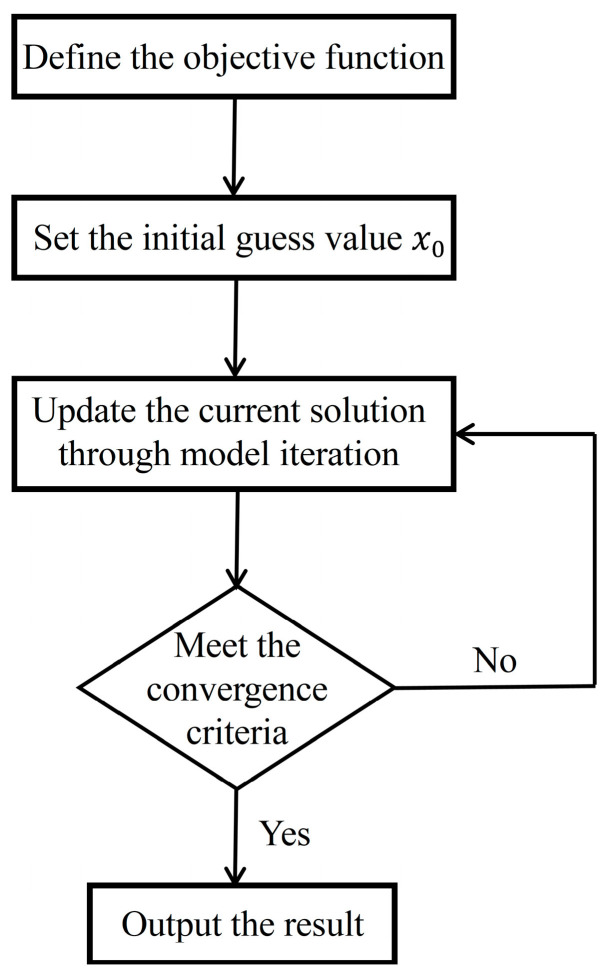
The flowchart of the inversely calculation of the corrected impedance.

As the characteristics of the transmission cable are known, the corrected impedance $Z_{c}$ can be solved from $Z_{x}$. The flowchart of the calculation is shown in Figure 4. First, the objective function is defined, and an initial guess value is assigned to initiate the iterative process. Subsequently, the current solution is updated iteratively by substituting the guess value into the equivalent circuit model. After each iteration, the updated solution is evaluated against predefined convergence criteria. If the solution does not meet these criteria, the process returns to further iterations to refine the solution. This iterative procedure continues until the convergence criteria are satisfied. Once convergence is achieved, the final output ${\;Z}_{c}$ represents the result obtained through reverse calculation.

### 2.3. Parameter Separation and Oxide Film Thickness Evaluation

**Figure 5:**
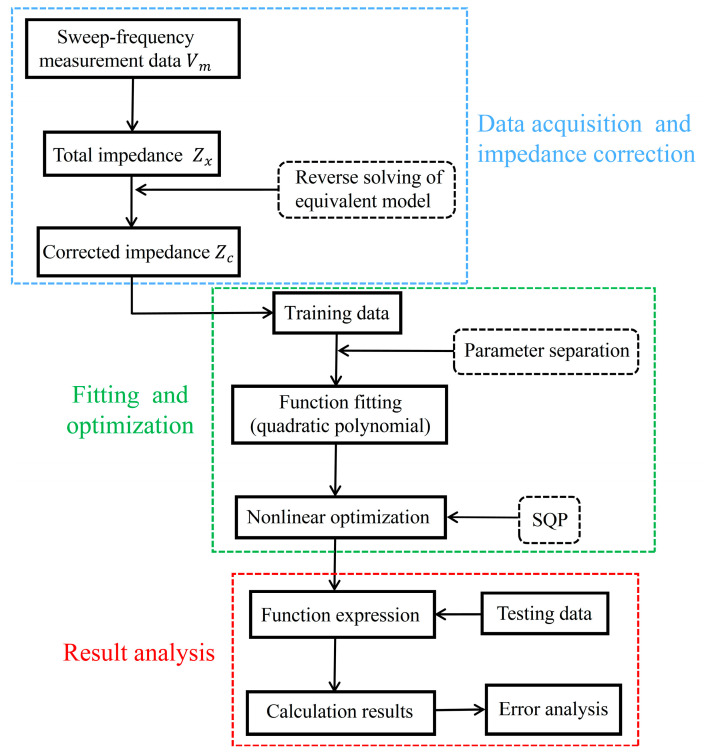
The diagram of the thickness measurement algorithm.

This paper develops an algorithm for quantifying oxidation film thickness based on swept frequency ECT. The algorithm utilizes a multi-parameter separation technique to mitigate the effects of other parameters, such as the conductivity of the substrate material. Figure 5 presents the diagram of the thickness measurement algorithm, which comprises three steps: data acquisition and impedance correction, fitting and optimization, and result analysis.

Firstly, swept frequency ECT is conducted on samples with different conductivities and oxide film thickness. The impedance $Z_{x}$ is calculated based on Equation (8), then the corrected impedance $Z_{c}$ is obtained by inverse calculation.

Secondly, the adjusted impedance is used to develop a mathematical model that delineates the correlation between impedance and oxide layer thickness. A quadratic polynomial is employed to fit this relationship, as shown in (10), where *d* is the thickness of the oxide film,${\;Z}_{cre\;}$and $Z_{cim}$ denote the real and imaginary components of $Z_{c}$, respectively, and${\;k}_{i}$ (i = 1, 2, … 5) are the coefficients. To minimize the influence of conductivity, the function must satisfy the constraint shown in (11), which indicates that the partial derivative of d with respect to conductivity σ should be minimized. The coefficients are calculated according to (13), where $k=[k_{1}\;k_{2}\;k_{3}\;k_{4}\;k_{5}]$ is the vector of the coefficients. The Sequential Quadratic Programming (SQP) approach is utilized to optimize the function fitting equation. The SQP algorithm is proficient at managing nonlinear constraints and improving the precision of intricate system models, thereby enabling accurate computation of the functional relationship between corrected impedance and oxidation film thickness [32].

(10) $$d=k_{0}+k_{1}\cdot Z_{\;cre\;}+k_{2}\cdot Z_{Cim}+k_{3}\cdot Z_{cre\;}^{2}+k_{4}\cdot Z_{cim\;}^{2}+k_{5}\cdot Z_{cre\;}Z_{cim}$$

(11) $$min|\frac{\partial d}{\partial \sigma }|$$

(12) $$\begin{array}{c} \frac{\partial d}{\partial \sigma }=k_{1}\frac{\partial Z_{cre}}{\partial \sigma }+k_{2}\frac{\partial Z_{cim\;}}{\partial \sigma }+2k_{3}\cdot Z_{cre\;}\cdot \frac{\partial Z_{c\;re\;}}{\partial \sigma }+2k_{4}\cdot Z_{c\;cim\;}\cdot \frac{\partial Z_{cim}}{\partial \sigma }\\ +k_{5}(Z_{cre}\cdot \frac{\partial Z_{cim}}{\partial \sigma }+Z_{cim}\cdot \frac{\partial Z_{cre}}{\partial \sigma }) \end{array}$$

(13) $$\underset{k}{min}|\frac{\partial d}{\partial \sigma }|$$

Thirdly, the independent test dataset is substituted into the fitted function expression to calculate oxide film thickness. Subsequently, error analysis is conducted on the results to assess the accuracy and reliability of the algorithm.

## 3. Experimental Setup

### 3.1. Probe Design

**Figure 6:**
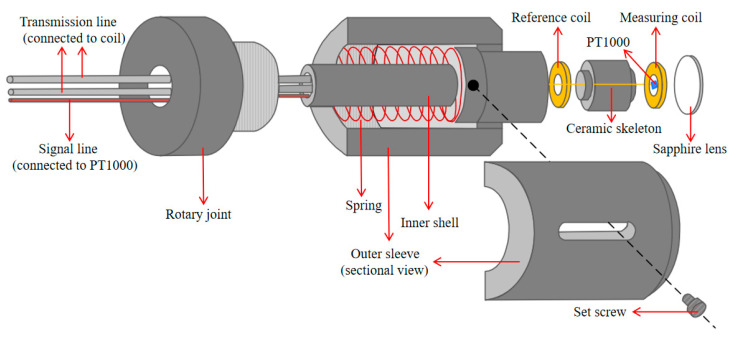
The schematic diagram of the probe.

The structure of the probe is illustrated in Figure 6. It consists of two coils and a PT1000 thermistor (Heraeus, Hanau, Germany). A spring mechanism is integrated into the design, applying consistent pressure on the inner shell to ensure stability and maintain firm contact between the probe and the surface of the fuel rod. The measuring coil is protected by a durable, heat-resistant sapphire lens, which is crucial for allowing the probe to glide smoothly along the surface of the fuel rod. The PT1000 sensor monitors the temperature, enabling real-time temperature compensation during measurements. The parameters of the coil are listed in Table 1.

**Figure 7:**
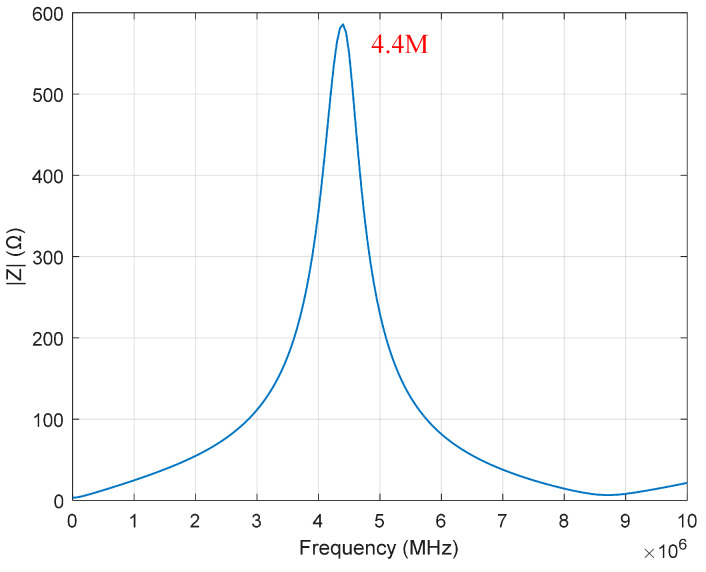
Frequency characteristic curve of the coil and the cable.

The length of the cable is 10 m. The frequency characteristic of the coil and the cable is measured using an impedance analyzer E4990A (Keysight Technologies, Santa Rosa, CA, USA). The frequency characteristic curve is depicted in Figure 7.

### 3.2. Experimental System

**Figure 8:**
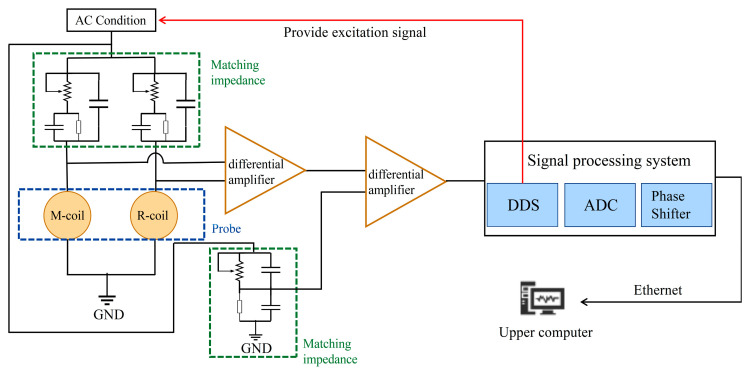
Schematic diagram of the experimental system.

The diagram of the experimental system is shown in Figure 8. The experimental system consists of a measuring bridge, a signal amplification circuit, a signal-processing system, and a host computer. The excitation signal is generated by the Direct Digital Synthesis (DDS) module within the signal-processing system. The measurement circuit employs a differential design to minimize the influence of environmental factors and the intrinsic effects of the probes. The differential amplifier processes the dual-ended signals from the probe, performing amplification and filtering to produce a single-ended measurement signal, which is subsequently sent to the lock-in amplifier. The lock-in amplifier isolates and extracts the signal component that matches the frequency of the excitation signal, effectively suppressing components at other frequencies. This ensures that the extracted signal is primarily derived from the induced current in the measurement coil. The output DC signal from the lock-in amplifier contains both the amplitude and phase information of the measurement signal, facilitating further processing. The real and imaginary components of the processed signal are transmitted to the computer for advanced data analysis, ensuring accurate and reliable interpretation of the measurement results.

**Figure 9:**
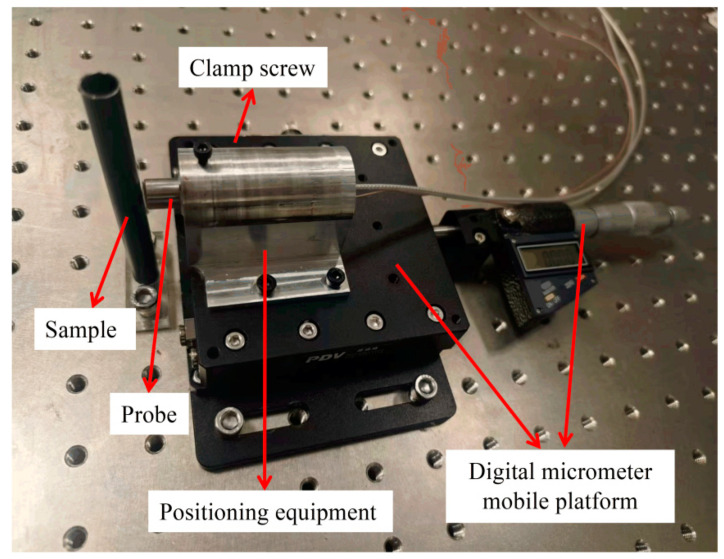
Photograph of the experimental apparatus.

Figure 9 presents a photograph of the experimental platform. The sample is firmly secured to an optical platform, while the movement of the mobile platform is regulated by a digital micrometer. This digital micrometer allows for precise control of the distance, achieving an accuracy of 0.1 μm, which ensures high precision in experimental positioning.

## 4. Results and Discussion

### 4.1. Experimental Results

The measurements were conducted at room temperature (approximately 25 °C) on five samples with varying conductivities, including 304 stainless steel, 316 stainless steel, brass, zirconium alloy, and aluminum alloy. Instead of using actual oxide films, the oxide film thickness was simulated by controlling the lift-off distance between the probe and the sample surface. This was achieved using a digital micrometer translation stage with a precision of 0.1 µm. The lift-off distance was adjusted in 10 µm intervals, covering a range from 0 to 70 µm, to mimic variations in oxide film thickness as per application requirements. The frequency range for the measurements was set from 0.4 MHz to 4.2 MHz, with a frequency step of 0.2 MHz. The data were subsequently processed using the proposed model to inversely calculate the corrected impedance ${\;Z}_{c}$, which was then incorporated into the algorithm to determine the oxide film thickness.

**Figure 10:**
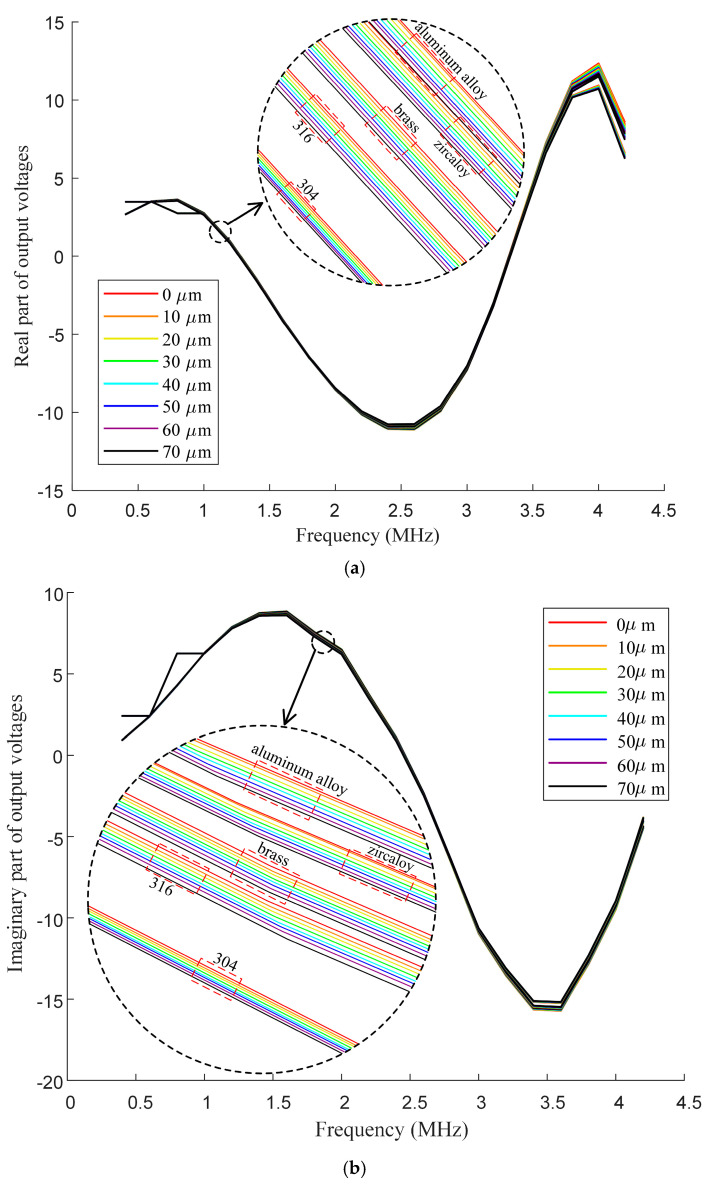
Experiment results: voltage ($V_{m}$) vs. frequency, (**a**) in−phase component and (**b**) quadrature component.

Figure 10 illustrates the in-phase and quadrature components of voltage $V_{m}$, measured at frequencies ranging from 0.4 MHz to 4.2 MHz for the five samples with varying electrical conductivities. In the figure, different colors are used to distinguish the various oxide film thicknesses. It seems that the recorded voltage is not monotonously correlated to the frequency, which is not suitable for the swept frequency technique. This is due to the effects of the cable.

**Figure 11:**
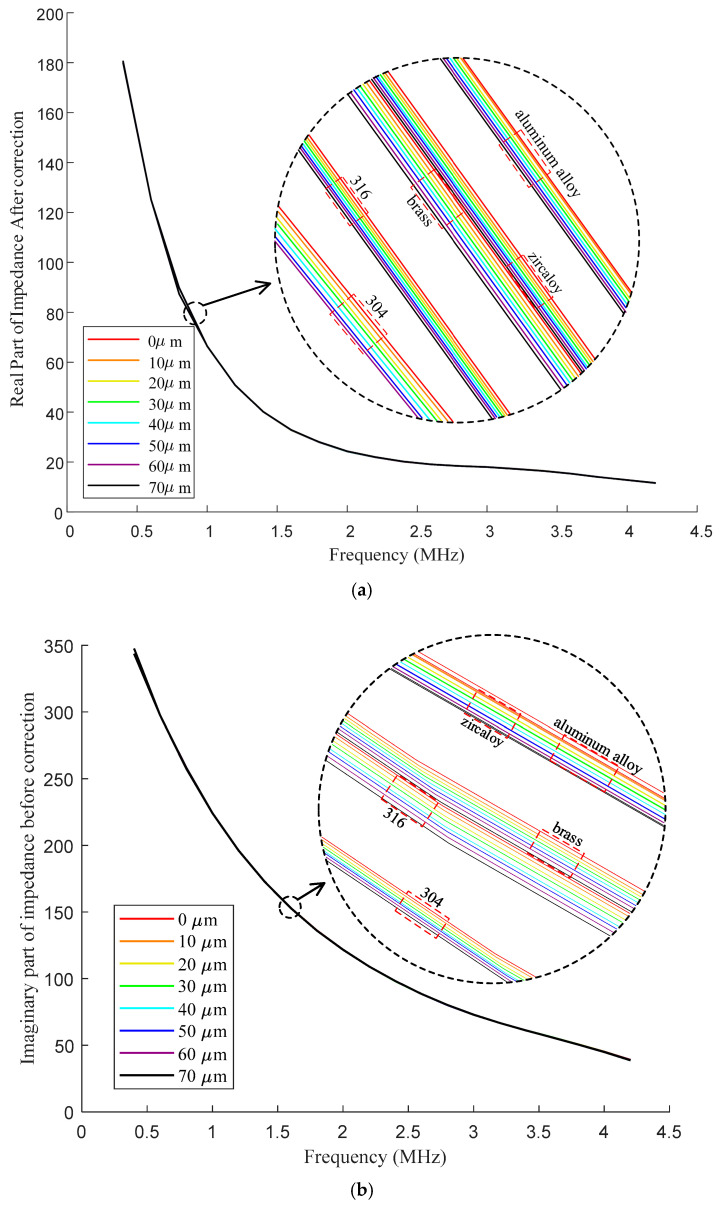
In−phase (**a**) and quadrature (**b**) components of impedance $Z_{c}$ after impedance correction.

Figure 11 depicts the in-phase and quadrature components of corrected impedance $Z_{c}$ derived from the data presented in Figure 10. It is seen that the curves become monotonous as the frequency varies. The corrected impedance values consistently decrease as the frequency increases. The impedance correction effectively mitigates the influence of the transmission cable. For samples with different electrical conductivities, the corrected impedance exhibits a negative correlation with oxide film thickness. Specifically, as the oxide film thickness increases, both the in-phase and quadrature components of the corrected impedance decline.

**Figure 12:**
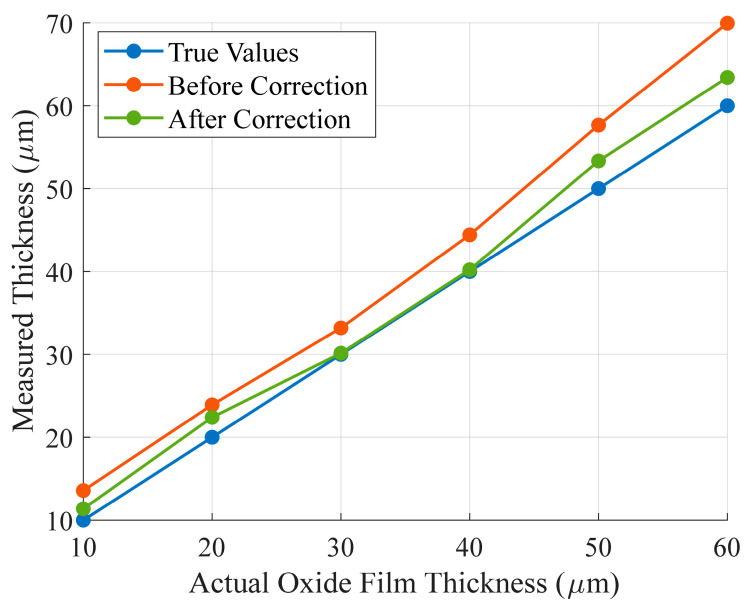
Comparison of thickness measurement results of zirconium alloy samples before and after impedance correction.

To evaluate the impact of impedance correction on accuracy, a comparative analysis was conducted by examining the data before and after the application of impedance correction. The data were processed by subtracting the baseline measurement taken with a 0 μm oxide film thickness. Figure 12 compares the thickness of the zirconium alloy samples derived from the data before and after impedance correction. Given that the cladding tube material is generally zirconium alloy, measurement data from four materials—304 stainless steel, 316 stainless steel, brass, and aluminum alloy—were utilized for curve fitting, while the measurement data from the zirconium alloy samples were employed as the test set. Various fitting functions were developed utilizing the data prior to and subsequent to impedance correction. Subsequently, the measurement data of the zirconium alloy samples were incorporated into these fitting functions for validation, resulting in the thickness measurement outcomes of the zirconium alloy samples prior to and following impedance correction. In Figure 12, the blue curve represents the actual oxide film thickness, the red curve represents the thickness calculated without impedance correction, and the green curve represents the thickness calculated with the corrected impedance. The results demonstrate that the thickness derived from the corrected impedance aligns more closely with the true value, demonstrating the effectiveness of the method.

Table 2 presents the absolute error of the oxide film thickness of the zirconium alloy samples before and after impedance correction. It was found that the maximum error decreased from 9.95 μm to 3.42 μm by using the impedance correction, and the average error decreased from 5.46 μm to 1.82 μm. The average relative error was reduced from 18.18% to 6.53%. These findings indicated a significant improvement in accuracy following impedance correction.

### 4.2. Discussion

The results indicate that, despite carrying out function fitting on materials with diverse conductivities, such as 304 stainless steel, 316 stainless steel, brass, and aluminum alloy, the algorithm effectively detects oxide layer thickness on zirconium alloy cladding tubes. The algorithm processes signals within the frequency range of 0.4 MHz to 4.2 MHz, which minimizes the effects of conductivity fluctuations by utilizing frequency response characteristics and constraint criteria. Therefore, regardless of the conductivity variance between the calibration sample and the test specimen, accurate readings can still be produced. The technique mitigates the impact of substrate conductivity fluctuations, hence guaranteeing dependable and precise oxide film thickness assessments.

One major challenge in accurately measuring oxide film thickness is the sensitivity of the sensor to temperature variations, commonly referred to as the temperature drift issue [33,34]. When nuclear fuel assemblies are removed from the reactor and transferred to the spent fuel pool for safety inspections, they retain elevated temperatures due to incomplete cooling. These temperature fluctuations significantly impact ECT sensor performance and may lead to measurement deviations. The influence of temperature changes on ECT signals is primarily reflected in the variation of electromagnetic parameters, such as the performance of the eddy current sensor and the electrical conductivity of the test material. These factors are often nonlinear and coupled. The Idaho National Laboratory in the United States has studied the performance of probes for measuring oxide film thickness on nuclear fuel cladding, evaluating the impact of temperature on eddy currents and measurement results. Their findings demonstrated a strong correlation between temperature variations and the detection results of eddy current sensors, ultimately concluding that the measured oxide film thickness decreases as the temperature increases [35]. Several studies on temperature compensation have aimed to address this issue, including the use of embedded temperature sensors [36], designing self-compensating circuits [37], developing high temperature-resistant materials [38], and applying appropriate compensation algorithms [39,40]. The focus of this paper is on eliminating the impact of transmission lines, and all experiments were conducted at approximately 25 °C (room temperature) without experimental investigation into temperature effects. In the future, we will conduct research on temperature compensation. By developing a model based on experimental data, we aim to effectively reduce the influence of temperature variations on ECT signals and improve the accuracy of oxide film thickness measurements.

## 5. Conclusions

This paper introduces a technique for precisely detecting oxide film thickness through a swept frequency approach and transmission cable impedance calibration process. The signals are calibrated based on a transmission line model of the cable, thus eliminating the influence of the transmission cable. The technology encompasses the development of a bespoke dual-coil probe, an impedance correction method utilizing a transmission line model, and a frequency sweep signal-processing algorithm, which successfully mitigates the effect of the transmission cable on high-frequency measurement outcomes. The parameter separation algorithm reduces the impact of differing substrate conductivities on measurement outcomes. A prototype probe has been developed, and an experimental platform has been established, incorporating a digital micrometer that offers precision of up to 0.1 microns. The experimental results indicated that, despite conducting function fitting with samples of differing conductivities (304 stainless steel, 316 stainless steel, brass, and aluminum alloy), the algorithm, following impedance correction, assessed the oxide film thickness on the zirconium alloy cladding surface with an average absolute error of merely 1.82 microns, a maximum absolute error of 3.42 microns, and an average percentage error of 6.53%. The findings demonstrate that the approach successfully reduces the effects of fluctuations in substrate conductivity. In comparison to pre-impedance correction, the average absolute error diminished by 66.7%, the maximum absolute error reduced by 65.6%, and the average percentage error decreased by 64.1%. This method is suitable for quantifying oxide coating thickness on nuclear fuel cladding and in various other applications. Future comprehensive research is essential to further advance the application and development of this technology. This includes investigating the impact mechanisms of temperature on measurement accuracy and developing robust temperature compensation methods. Additionally, further studies should focus on examining the effects of weakly magnetic surface scaling and exploring methods for accurately measuring the thickness of scale layers. Other areas of research include the examination of longer transmission cables to improve signal integrity, the enhancement of algorithms to optimize detection performance, and the integration of advanced techniques to address environmental and operational challenges.

## Figures and Tables

**Table 1:** Parameters of the coils.

Parameter	Value
Inner diameter	4.1 mm
Outer diameter	5.6 mm
Height	0.5 mm
Wire diameter	0.1 mm

**Table 2:** Comparison of errors in oxide film thickness measurements of zirconia alloy samples before and after impedance correction. (Unit: μm).

Actual Value	Before Impedance Correction	After Impedance Correction
10	3.58	1.37
20	3.92	2.41
30	3.20	0.15
40	4.43	0.22
50	7.67	3.35
60	9.95	3.42

## Data Availability

The data presented in this study are available upon request from the corresponding author.
